# Trait Values, Not Trait Plasticity, Best Explain Invasive Species' Performance in a Changing Environment

**DOI:** 10.1371/journal.pone.0048821

**Published:** 2012-10-31

**Authors:** Virginia Matzek

**Affiliations:** Department of Environmental Studies and Sciences, Santa Clara University, Santa Clara, California, United States of America; University of Konstanz, Germany

## Abstract

The question of why some introduced species become invasive and others do not is the central puzzle of invasion biology. Two of the principal explanations for this phenomenon concern functional traits: invasive species may have higher values of competitively advantageous traits than non-invasive species, or they may have greater phenotypic plasticity in traits that permits them to survive the colonization period and spread to a broad range of environments. Although there is a large body of evidence for superiority in particular traits among invasive plants, when compared to phylogenetically related non-invasive plants, it is less clear if invasive plants are more phenotypically plastic, and whether this plasticity confers a fitness advantage. In this study, I used a model group of 10 closely related *Pinus* species whose invader or non-invader status has been reliably characterized to test the relative contribution of high trait values and high trait plasticity to relative growth rate, a performance measure standing in as a proxy for fitness. When grown at higher nitrogen supply, invaders had a plastic RGR response, increasing their RGR to a much greater extent than non-invaders. However, invasive species did not exhibit significantly more phenotypic plasticity than non-invasive species for any of 17 functional traits, and trait plasticity indices were generally weakly correlated with RGR. Conversely, invasive species had higher values than non-invaders for 13 of the 17 traits, including higher leaf area ratio, photosynthetic capacity, photosynthetic nutrient-use efficiency, and nutrient uptake rates, and these traits were also strongly correlated with performance. I conclude that, in responding to higher N supply, superior trait values coupled with a moderate degree of trait variation explain invasive species' superior performance better than plasticity per se.

## Introduction

Invasive species have long proved puzzling to the ecologist: Why do some species become invasive outside their native range, and others do not? One line of reasoning common to many investigations of invasive plants is that invaders have particular traits that make them superior competitors in the invaded habitat. Invasive species may possess novel traits that are poorly represented in the native flora, such as N fixation [1], or they may exhibit more extreme values of competitively advantageous traits than do the local species [2]–[4]. Multispecies studies comparing phylogenetically related invaders to non-invaders have begun to yield insights into which traits are typically associated with invasion success, which may boost efforts to screen plants for invasiveness before introduction [5]–[8].

A separate line of reasoning with regard to plant traits is that invaders have higher phenotypic plasticity, which has long been theorized to promote invasion by permitting introduced species to colonize a broader range of environments, or escape extinction in the early period of invasion when the number of available genotypes is small [9]–[11]. Empirical studies comparing plasticity in invasive and non-invasive plants are now so numerous that they have been subjected to meta-analysis twice–but the meta-analyses came to different conclusions [12], [13], with one concluding that invaders showed higher plasticity and the other finding no evidence for such a trend. Likewise, plasticity may be adaptive if it increases fitness (or permits smaller declines in fitness in response to harsher conditions) [14], [15], but whether higher plasticity has resulted in higher fitness or invasion success in invasive species has not been made entirely clear by literature reviews or by models [13], [16], [17].

Studies of phenotypic plasticity have special significance for understanding the response of known invaders to global change. The possible shrinkage or expansion of invaders' range as a consequence of nitrogen deposition, climate warming, increased atmospheric CO_2_, or other aspects of environmental change is an issue of serious consequence to land and resource managers, and these responses may be partly mediated by plasticity [18]–[20]. It would therefore be useful to have more multispecies, phylogenetically controlled comparisons to evaluate the relative contribution of competitively advantageous traits, and plasticity in those traits, as mechanisms of invasion success. As several authors have pointed out [8], [13], [14], [21], even low plasticity may be adaptive in a species that has high values of traits that confer a competitive advantage. Unfortunately, comparisons between invaders and their native or non-invasive counterparts have been performed according to a wide variety of (sometimes problematic) experimental designs [7], including comparing invaders and native species of radically different phylogenetic history, or comparing invaders to indigenous species that have never been introduced outside their native range and therefore have unknown potential for invasiveness [22].

In this study, I investigate the trait values and trait plasticity of a group of 10 species of known invasiveness, all in the genus *Pinus,* grown at different levels of nitrogen fertilization. *Pinus* has been suggested as an ideal model for invasion studies because pine species of tremendous ecological variety have been widely introduced and invasiveness (or lack thereof) is well documented for many members of the genus [23]. In lieu of a measure of reproductive fitness, which was impractical to consider in these long-lived trees, I used the relative growth rate, a performance measure which encompasses many aspects of plant function and has important effects on competitive ability and recruitment [24]. Proxies for fitness relying on biomass or size are common in plasticity studies because of the practical difficulty of measuring fitness [25]. To generalize within this group of closely related congeners, I examined species-level plasticity–i.e., plasticity expressed across an environmental gradient by individuals from the same population [15], as opposed to genotype-level plasticity, the expression of different phenotypes in different environments by a single genotype [9]. I sought to answer the following questions:

1. How do invasive pines compare to non-invasive pines in functional traits at different levels of N supply?
2. Are invasive species more plastic than non-invasive species in responding to increased N? Which traits are most plastic as N supply changes?
3. Which trait values correlate with the fitness proxy, RGR? For which traits does phenotypic plasticity itself appear to be adaptive?

## Methods

### Experimental design

Study species comprised five invasive pines (*Pinus banksiana, P. halepensis, P. muricata, P. pinaster,* and *P. radiata*) and five non-invasive pines (*P. cembra, P. flexilis, P. lambertiana, P. sabiniana,* and *P. torreyana*). “Invasive” pines have a record of invasiveness on at least two continents, while “non-invasive” pines have no reports of invasiveness after planting on at least three continents [2], [26]. Most of the species are in the subgenus *Pinus*, but two of the non-invaders, *P. cembra* and *P. flexilis*, are in the subgenus *Strobus* and are therefore more distantly related to the others [27].

Pine seeds obtained from commercial suppliers were germinated in a sand-vermiculite mixture after a species-specific cold stratification period [28]. Seedlings were transplanted into nutrient treatments when their second set of true leaves emerged, to minimize ontogenetic differences between species. To avoid effects of environmental gradients within the greenhouse, the experiment was blocked, with each of the 9 blocks containing both nutrient treatments and 1 randomly selected individual of each species per treatment. Nine seedlings of each species (except *P. cembra*; n = 7) were grown in 35L pots with nitrogen supply of 50 mg N pot^−1^ wk^−1^ (high N treatment) or 1 mg N pot^−1^ wk^−1^ (low N treatment). Phosphorus was supplied at 10 mg P pot^−1^ wk^−1^ and all other nutrients were supplied in abundance as a half-strength N- and P-free Hoagland's solution. Seedlings grew for 12 weeks after transplant into the treatments. Plants were watered freely and monitored with a soil probe to ensure that moisture was not limiting to plant growth. Average midday PAR at plant height was approximately 1350 μmol/m^2^/s. Greenhouse temperatures were ∼25°C (day) and ∼15°C (night), with daylength set at 12 h.

Twenty additional seedlings of each species (except *P. cembra*; n = 13) were randomly selected for destructive harvest at transplant size and used to estimate initial seedling weight for calculation of the relative growth rate (RGR; total plant dry biomass per unit initial seedling dry weight per day) and the specific absorption rate (SAR; net gain of nutrient per unit root mass per day), integrated over the harvest interval of 12 weeks [29].

### Measurement of physiological and morphological traits

Seventeen traits related to biomass allocation, resource capture, leaf construction costs, and nutrient use and uptake efficiency were selected for analysis, because variation in these traits may confer fitness advantages, and because they provide useful points of comparison to other plasticity studies. The photosynthetic rate of each individual was measured immediately prior to final harvest by gas exchange, on a detached shoot tip enclosed in the conifer chamber of a portable infrared gas analyzer (LiCor LI-6400, Lincoln, NE). Measurements were made on several different days outdoors in full sun, when temperatures were moderate (20–30°C), relative humidities were in the range of 70%–75%, and light intensities ranged from 1400–1600 μmol/m^2^/s. CO_2_ input was fixed at 400 ppm and airflow through the chamber was 500 μmol/s. Measurements were made as soon as the CO_2_ concentration in the chamber stabilized, typically <2 minutes. Self-shading was minimized by orienting the chamber so that the shoot tip was maximally illuminated. The maximal photosynthetic rate was calculated on an area basis using a leaf mass/area conversion (methods below). Photosynthetic nitrogen-use efficiency (PNUE) was calculated as the ratio of A_area_ and N_area_ (μmol CO_2_ g N^−1^ s^−1^), using the photosynthetic rate of individuals in each species and the value for N or P concentration from the species tissue composite (below). Instantaneous water-use efficiency (WUEi) was calculated as the ratio of photosynthesis to transpiration (μmol CO_2_ mmol H_2_O^−1^ s^−1^).

Chemical analyses were performed on the two youngest fully expanded whorls of needles at each shoot tip, which were removed, weighed, and flash frozen in liquid nitrogen at the time of harvest. The frozen leaf tissue was bulked by species, ground in liquid nitrogen with a mortar and pestle, and either stored at −80°C for use in protein and chlorophyll measurements, or oven-dried at 55°C for assays of N and P concentration by Kjeldahl digest. The remaining root, stem, and leaf tissue were separately weighed and oven-dried at 55°C for measurement of biomass allocation traits (LMR =  leaf mass ratio, SMR  =  stem mass ratio, RMR  =  root mass ratio, LAR  =  leaf area ratio) and leaf dry mass fraction (DMF, ratio of dry mass to fresh mass). Specific leaf area (SLA) was calculated from the projected leaf area of fresh needles from a subset of 3–4 harvested individuals that were scanned on a flatbed scanner before being dried and weighed. Protein, chlorophyll, and nutrient content results are expressed on an area basis.

Soluble protein content was determined by a Lowry assay compatible with detergents and reducing agents, using an extract of frozen leaf tissue heated to 55°C in a buffer of 5% sucrose, 5% sodium dodecyl sulfate, and 5% β-mercaptoethanol [30]. For chlorophyll measurements, absorbance of an extract of frozen leaf tissue in 100% acetone was measured at 662 and 645 nm to determine chl *a* and *b* concentrations [31].

### Data analysis

To increase the generalizability of the results, and because the small amount of biomass available from individuals in the low-nutrient treatment required bulking tissue from different individuals into a species composite, species is the replicate for this study, not individuals. Mean trait values for all 17 measured traits, plus RGR, were calculated for each species in each nitrogen treatment. To assess the effects of nitrogen availability and invasiveness on plant traits, I performed a mixed-model, nested ANOVA with “nitrogen level” and “invasive status” as fixed effects and “species” nested in “invasive status” as a random effect, for all traits except biomass allocation traits. Biomass allocation patterns can be influenced by plant size, so leaf-, stem-, and root-mass ratios (LMR, SMR, and RMR, respectively) were analyzed as mixed-model nested ANCOVAs with final harvest biomass as a covariate. Because species is the replicate in this experiment, variation among individuals of a species and among greenhouse blocks does not figure into the ANOVA and ANCOVA analyses. Using species as a replicate comes at a sacrifice of some statistical power, but has the advantage of using the same level of data resolution for the trait value analysis as the plasticity index analysis (below).

To quantify trait plasticity, I calculated a plasticity index (PI_v_) [32] as the difference between the maximum and minimum values of the trait mean, divided by the maximum value, for each species. There are many different ways to calculate relative plasticity [33]; this one approximates a reaction norm and has the advantage of being insensitive to differences in variance between samples in the two environments [21], [32]. Student's t-test for unequal variances was used to distinguish invader and non-invader groups for each trait.

Though useful for calculating averages and correlations, a disadvantage of an absolute-value measure like PI_v_ is that it obscures the direction of the response. Invasive species and non-invasive species may differ in whether they increase or decrease trait values in response to increased N. Therefore, a second plasticity index, the relative trait range (RTR) [34], was calculated to see whether systematic differences existed between invaders and non-invaders in the sign of change for any trait. To determine the RTR, I subtracted the mean trait value in the low N treatment from the mean trait value in the high N treatment and divided it by the maximum of these two values. Positive RTR values mean that the trait value increased in response to higher N supply. RTR values were not used in statistical calculations, but are identical to PI_v_ values except for sign.

The relative growth rate (RGR) under high resource conditions was used as a measure of performance and a proxy for fitness. For each trait, I evaluated whether species' mean trait values across nutrient treatments, or their mean plasticity indices, were significantly correlated with mean RGR across treatments, by calculating Pearson product-moment correlation coefficients for the association.

Because analysis of 17 separate traits involves performing multiple comparisons on the same dataset, it is necessary to adjust the probability of Type I error for the large number of statistical tests. Rather than using the sequential Bonferroni correction, which has the drawback of greatly inflating the probability of Type II error, I instead report all exact p-values and control the false discovery rate using the procedure of Benjamini and Hochberg [35], which is suitable when tested variables lack independence from each other [36], as is true here.

## Results

### Comparison of mean trait values for invasive and non-invasive pines at different N levels

**Figure 1:**
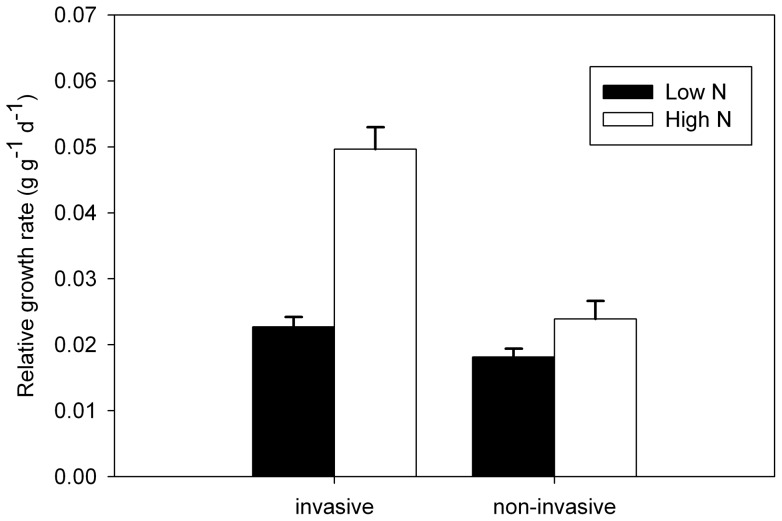
Performance (RGR) of invasive and non-invasive species across nutrient treatments. Values are means ± standard error of invasive and non-invasive species groups in low-N and high-N treatment.

The N treatments had a strong effect on plant performance, as represented by relative growth rate (RGR). Relative growth rate was increased both by high nitrogen and invasive status, and there was a significant interaction whereby invasive species benefited more from the high nitrogen levels than did non-invaders in increasing their growth rate (Figure 1).

Descriptions and units for the 17 functional traits measured in the experiment are shown in Table 1. All of the biomass allocation traits differed significantly between the invasive group and the non-invasive group (Table 2 and 3). Invaders had higher leaf mass ratio (LMR) and leaf area ratio (LAR), but lower root mass ratio (RMR) and stem mass ratio (SMR), than non-invaders. Nitrogen level also affected allocation to biomass; increased nitrogen caused pine seedlings to increase leaf mass and leaf area ratios, and decrease root mass ratios.

**Table 1:** Descriptions of traits and performance measure (fitness proxy).

Abbreviation	Description	Units
RGR	Relative growth rate	g plant g^−1^ init wt d^−1^
LMR	Leaf mass ratio	g leaf g^−1^ plant
RMR	Root mass ratio	g root g^−1^ plant
SMR	Stem mass ratio	g stem g^−1^ plant
LAR	Leaf area ratio	cm^2^ leaf g^−1^ plant
SLA	Specific leaf area	cm^2^ mg^−1^ leaf
DMF	Dry mass/fresh mass ratio	
P_area_	Phosphorus content per unit area	g p m^−2^ leaf
N_area_	Nitrogen content per unit area	g N m^−2^ leaf
chl_area_	Chlorophyll (a+b) content per unit area	μg chl cm^−2^ leaf
protein_area_	Protein content per unit area	mg protein cm^−2^ leaf
A_area_	Photosynthetic rate per unit area	μg mol CO_2_ m^−2^ leaf s^−1^
g_s_	Stomatal conductance	mol CO_2_ m^−2^ leaf s^−1^
PNUE	Photosynthetic nitrogen-use efficiency	mmol CO_2_ g N^−1^ s^−1^
WUEi	Instantaneous water-use efficiency	mmol CO_2_ mmol^−1^ H_2_O s^−1^
SAR_nitrogen_	Specific absorption rate of N	mg N gain g^−1^ root d^−1^
SAR_phosphorus_	Specific absorption rate of P	mg P gain g^−1^ root d^−1^

**Table 2:** Trait values for invasive and non-invasive species at two nitrogen levels.

Trait	Low nitrogen		High nitrogen	
	*invasive*	*non-invasive*	*invasive*	*non-invasive*
LMR	505±013(a,b)	452±030(a)	583±014(c)	510±025(b)
RMR	348±022(a)	375±027(a)	256±013(c)	308±023(b)
SMR	172±013(b)	193±024(a,b)	188±012(a,b)	199±023(a)
LAR	21.020±1.890(b)	14.290±1.185(c)	23.913±1.124(a)	16.275±1.379(b,c)
SLA	42.519±3.494(a)	32.319±2.238(b)	42.112±2.089(a)	32.669±2.359(b)
DMF	255±016(b)	303±016(a)	201±007(c)	262±022(a,b)
P_area_	1.359±248(a)	1.196±132(a)	1.213±129(a)	1.312±140(a)
N_area_	3.327±305(c)	5.051±1.059(b)	6.681±841(a,b)	8.182±1.373(a)
chl_area_	19.57±2.33(a)	33.11±11.95(a)	41.07±6.67(a)	43.97±4.43(a)
Chl a/b	3.342±090(a)	3.472±141(a)	3.704±189(a)	3.441±106(a)
protein_area_	1.109±266(a)	1.406±393(a)	1.713±233(a)	1.361±179(a)
A_area_	16.795±1.740(a)	13.599±0.768(a)	34.761±4.442(b)	20.219±4.139(a,b)
g_s_	229±034(b)	191±038(b)	449±077(a)	262±068(a,b)
PNUE	5.090±368(a)	3.142±647(a)	5.557±1.086(a)	2.723±569(a)
WUEi	1.769±214(b)	2.899±518(a)	2.386±205(a,b)	2.732±350(a)
SAR_nitrogen_	807±252(b)	136±029(b)	5.993±697(a)	2.056±326(b)
SAR_phosphorus_	840±190(a)	551±193(a)	2.856±266(b)	1.179±460(a)

Trait abbreviations as in Table 1. Values are means ± standard errors. Different lower-case letters within rows denote significant differences (α = .05) from mixed-model, nested ANOVAs using “nitrogen level” and “invasive status” as fixed effects and “species” nested in “invasive status” as a random effect. Biomass allocation traits (top 4 rows) were analyzed as mixed-model nested ANCOVAs with final harvest biomass as a covariate.

**Table 3:** ANOVA and ANCOVA statistics for trait values.

Trait	Nutrient		Status		Nutrient × Status	
	*F*	*p*	*F*	*p*	*F*	*p*
LMR	19.2101	**0.0032**§	69.4507	**<.0001**§	0.9853	0.354
RMR	29.9966	**0.0009**§	19.1447	**0.0333**§	2.8382	0.1359
SMR	4.2337	0.0786	13.6685	**0.0077**§	1.9603	0.2042
LAR	14.5617	**0.0066**§	115.4058	**<.0001**§	3.493	0.1038
SLA	0.0002	0.9878	29.0339	**0.0007**§	0.0433	0.8404
DMF	26.8405	**0.0008**§	35.6139	**0.0003**§	0.5429	0.4823
P_area_	0.0075	0.9332	0.0337	0.859	0.5677	0.4728
N_area_	48.8286	**0.0001**§	12.0779	**0.0084**§	0.0577	0.8163
chl_area_	8.5546	**0.0191**§	2.2099	0.1754	0.9245	0.3645
Chl a/b	1.2486	0.2963	0.2029	0.6644	1.7647	0.2207
protein_area_	1.4839	0.2579	0.0141	0.9083	1.9935	0.1957
A_area_	12.606	**0.0075**§	6.5621	**0.0336**§	2.6849	0.1399
g_s_	10.6068	**0.0116**§	6.3525	**0.0358**§	2.7816	0.1339
PNUE	0.0014	0.9709	14.4137	**0.0053**§	0.4958	0.5013
WUEi	1.7596	0.2213	18.9281	**0.0024**§	5.3531	**0.0494**
SAR_nitrogen_	67.8128	**<.0001**§	28.497	**0.0007**§	14.3216	**0.0054**§
SAR_phosphorus_	19.423	**0.0023**§	10.7436	**0.0112**§	5.3555	**0.0494**

Trait abbreviations as in Table 1. Test statistics are for mixed-model, nested ANOVAs using “nitrogen level” and “invasive status” as fixed effects and “species” nested in “invasive status” as a random effect, except biomass allocation traits (top 4 rows), which were analyzed as mixed-model nested ANCOVAs with final harvest biomass as a covariate. Boldface denotes p-values less than.05; § denotes p-values significant at α = .05 when corrected for 17 comparisons by the Benjamini-Hochberg procedure.

Of leaf-level traits, invasives had higher specific leaf area (SLA) and lower dry-mass fraction (DMF) than did non-invasives. Also, photosynthetic capacity (A_area_) and stomatal conductance (gs) were higher, but leaf nitrogen (Narea) was lower, in invaders than non-invaders. High N supply increased leaf nitrogen content, chlorophyll content (chl_area_), photosynthesis, and stomatal conductance, and decreased leaf DMF.

All the whole-plant traits associated with nutrient use and uptake showed greater efficiency in the invader group. However, the specific root absorption rates for nitrogen and phosphorus (SAR_nitrogen_ and SAR_phosphorus_) also were affected by N supply, with a significant interaction between invasive status and nitrogen level for SAR_nitrogen_ whereby invaders increased their N uptake rate to a greater degree than non-invaders when N supply increased. Photosynthetic nitrogen-use efficiency (PNUE) was increased only by invasive origin, not by nitrogen supply. Instantaneous water-use efficiency (WUEi) was higher in non-invaders than invaders, and was unaffected by N level.

**Figure 2:**
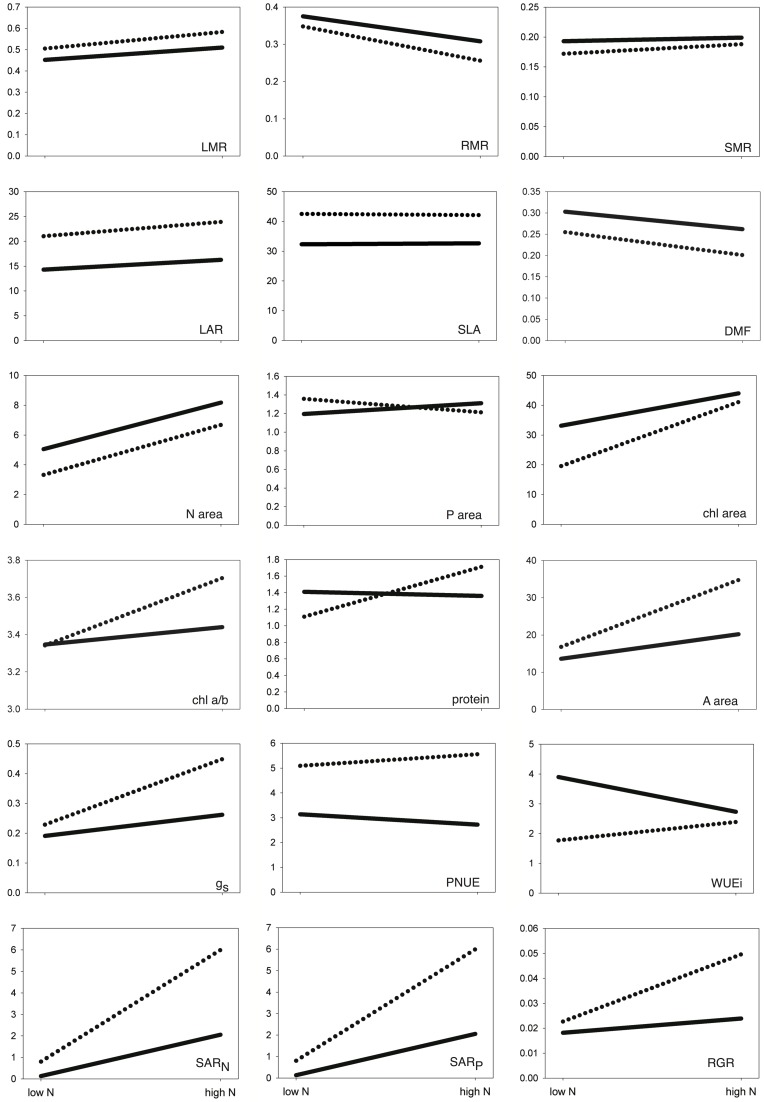
Trait reaction norms for invasive and non-invasive species across nutrient treatments. Dotted line  =  invasive species; solid line = non-invasive species. Trait abbreviations are as in Table 1. For each trait, the line links the mean in the low-N treatment to the mean in the high-N treatment, so steeper slopes indicate greater relative responses to the change in nutrient supply.

Reaction norms for traits of invaders and non-invaders responding to the increase in N supply are summarized in Figure 2. To see mean values for every trait in each species, consult the Supplemental Information (Table S1).

### Comparison of mean plasticity values for invasive and non-invasive pines

No significant difference in the plasticity index, PI_v_, between the groups of invasive species and non-invasive species was apparent for any trait, after correction for multiple comparisons (Table 4). Relative trait range index (RTR) values, which are identical to PI_v_ values except that they indicate (by their sign) whether trait values increased or decreased in response to higher N, are reported for all 10 species and 17 traits in the Supplemental Information (Table S2).

**Table 4:** Plasticity indices (PI_v_) for invasive and non-invasive species.

Traits	Invasive	Invasive response	Non-invasive	Non-invasive response	t	p
LMR	133±016	Increase	116±021	increase	0.6696	0.5231
RMR	260±032	Decrease	182±010	decrease	2.2934	0.0732
SMR	116±012	Mixed	041±015	mixed	3.86	**0.0053**
LAR	142±051	mixed	121±024	increase	0.3789	0.7186
SLA	0.134±093	mixed	037±037	mixed	1.46255	0.1437
DMF	205±03	decrease	135±10	decrease	2.161	0.0807
P_area_	281±058	mixed	276±055	mixed	1.26	0.2469
N_area_	489±037	increase	380±071	increase	0.0573	0.9557
chl_area_	483±084	increase	411±100	mixed	1.36	0.2224
Chl a/b	091±043	increase	102±031	mixed	0.5544	0.5949
protein_area_	395±106	mixed	323±134	mixed	0.2093	0.8399
A_area_	462±117	increase	324±072	mixed	0.4242	0.6832
g_s_	491±092	mixed	271±051	mixed	1.008	0.3488
PNUE	286+.072	mixed	209±071	mixed	0.7607	0.4687
WUEi	267±083	mixed	158±058	mixed	1.216	0.274
SAR_nitrogen_	856±049	increase	933±010	increase	1.57	0.1867
SAR_phosphorus_	733±078	increase	559±052	mixed	1.86	0.1149
all allocation	163±018		115±009		2.3623	0.0585
all leaf-level	337±026		251±023		2.4928	**0.0378**
all whole-plant	535±021		457±018		2.807	**0.0237**

**Figure 3:**
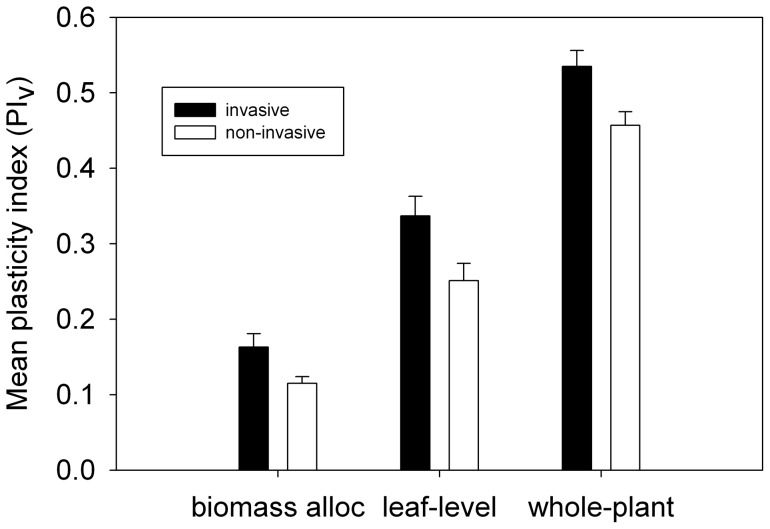
Differences in mean plasticity between invasive and non-invasive species by trait grouping. Values are means ± standard error for invasive and non-invasive species, where the plasticity indices of several traits in a grouping have been averaged together for each of the 5 invasive and 5 non-invasive species. For the biomass allocation grouping, each species' value is its mean plasticity index for the traits LMR, SMR, RMR and LAR; for the leaf-level grouping, each species' value is its mean plasticity index for the traits SLA, DMF, P_area_, N_area_, chl_area_, protein_area_, A_area_, and g_s_; and for the whole-plant grouping, each species' value is its mean plasticity index for the traits PNUE, WUEi, SAR_N_, and SAR_P_. After correction for multiple comparisons, no differences were statistically significant.

Trait abbreviations as in Table 1. Test statistics (t) and p-values (p) are from Student's t-test for unequal variances; p-values in boldface are <.05. The bottom three rows represent mean values for combined indices with several traits' plasticity indices averaged together by species (see also Figure 3). No plasticity indices for individual traits were significantly different between invasive and non-invasive groups after Benjamini-Hochberg correction for 17 trait comparisons, nor were grouped indices significantly different after correction for three comparisons. Means, standard errors, and test statistics were calculated using the PI_v_, which represents the absolute value of the change in trait value between N treatments, but a second index, the RTR, was used to determine the directionality of the response, which is indicated to the right of each PI_v_ column for individual traits. “Increase” means that all species in the group increased the trait value in response to increased N; “decrease” means that all species in the group decreased the trait value; and “mixed” means that at least one increase and one decrease were observed in the species group. Table S2 in the Supplementary Information shows RTR values for all traits and all species.

Generally, plasticity indices decreased in the order whole-plant > leaf-level > biomass allocation. The most plastic traits in response to nitrogen supply were those associated with nutrient uptake (SAR_nitrogen_ and SAR_phosphorus_), leaf nitrogen (N_area_ and chl_area_), and photosynthesis (A_area_ and g_s_). The least plastic traits were chlorophyll a/b ratio, stem mass ratio, and SLA (Table 4).

When trait plasticity indices were averaged together in groups of traits (biomass allocation: LMR, SMR, RMR, and LAR; leaf-level: SLA, DMF, P_area_, N_area_, chl_area_, protein_area_, A_area_, and g_s_; whole-plant: PNUE, WUEi, SAR_nitrogen_ and SAR_phosphorus_), greater plasticity in the invasive species was apparent for all the groupings, but still did not rise to the level of statistical significance after correction for three simultaneous comparisons (Table 4, Figure 3). Whole-plant trait plasticity was the most different between invasives and non-invasives, followed by leaf-level trait plasticity and then by plasticity in biomass allocation traits.

### Correlation with performance (RGR)

Trait values were much more frequently and strongly correlated with performance than plasticity indices (Table 5). Only the plasticity index for SMR was significantly correlated with RGR; however, closer examination of the data revealed that SMR plasticity for one species, *P. cembra,* was an extreme outlier (defined as <1.5 times the interquartile range for the distribution). Deletion of the outlying data point resulted in a non-significant correlation, though still a strong one (r = .76557, p = .0448). Among trait values in the biomass allocation group, LMR and LAR were significantly correlated with RGR, as were SLA and dry-mass fraction among the leaf-level traits. The strongest performance correlates were at the whole-plant level, where variation in PNUE and SAR_nitrogen_ and SAR_phosphorus_ each explained >80% of the variation in RGR.

**Table 5:** Correlation of trait values and plasticity indices with performance.

Trait	Trait value		Plasticity index	
	*r*	*P*	*r*	*p*
LMR	0.75556	**0.0115**§	−0.03584	0.9217
RMR	−0.54992	0.0996	0.53532	0.1108
SMR	−0.23861	0.5067	0.83433	**0.0027**§
LAR	0.90241	**0.0004**§	−0.08539	0.8146
SLA	0.80569	**0.0049**§	0.64762	**0.0429**
DMF	−0.80043	**0.0054**§	0.47629	0.164
N_area_	−0.63011	0.0509	0.54719	0.1016
P_area_	−0.28759	0.4204	−0.11245	0.7571
chl a/b	−0.02989	0.9347	−0.23981	0.5046
chl_area_	−0.57344	0.0831	0.40295	0.2483
protein_area_	−0.10995	0.7624	0.10118	0.7809
A_area_	0.63557	**0.0483**	0.57877	0.0796
g_s_	0.654	**0.0402**	0.75942	**0.0108**
PNUE	0.93944	**.0001**§	0.25531	0.4765
WUEi	−0.49952	0.1416	0.26716	0.4555
SAR_nitrogen_	0.87841	**0.0008**§	−0.46466	0.176
SAR_phosphorus_	0.75985	**0.0108**§	0.59434	0.07
all allocation			0.42409	0.2219
all leaf-level			0.84134	**0.0023**§
all whole-plant			0.57544	0.08176

Trait abbreviations as in Table 1. Pearson product-moment correlations (r) and p-values (p) for the relationship between mean trait values or mean plasticity index and mean RGR. Mean values are the average of the two nutrient treatments. The bottom three rows represent correlations between mean RGR and a combined plasticity index drawn from several traits (see text). Boldface denotes p-values less than.05; § denotes p-values significant at α = .05 when corrected for 17 multiple comparisons by the Benjamini-Hochberg procedure for individual traits or indices, and for 3 multiple comparisons for grouped plasticity indices. After elimination of an outlier for SMR plasticity (see text), the correlation coefficient r = .76557 and p = .0448 (non-significant).

When trait plasticity indices were combined according to the groupings above, leaf-level trait plasticity was significantly associated with RGR, but biomass allocation and whole-plant trait plasticity were not.

## Discussion

### Trait values

Thirteen of the seventeen traits measured in this study differed between invaders and non-invaders, and ran the gamut of plant function from biomass allocation and leaf morphology to nitrogen uptake and photosynthetic efficiency. In a recent meta-analysis of trait comparisons related to invasiveness, invaders were found to be significantly different from non-invaders in an equally wide range of functional categories: shoot allocation, leaf-area allocation, physiology, size, growth rate, and fitness [37].

Some of my results supported the idea that invaders tend to occupy the “quick-return” end of the so-called leaf economics spectrum [38], where high carbon fixation rates and nutrient contents are associated with shorter leaf lifespans and thinner, less dense leaves. Several studies have attributed higher SLA, LAR, leaf nutrients, and/or photosynthetic capacity to invasives, including a large-dataset study of local and global leaf traits [39] and some studies of invasive-native congener pairs [40]–[44]. Leaves with higher SLA and lower DMF present more surface area for gas exchange relative to their investment in biomass and construction costs, so this trait syndrome may confer a strong competitive advantage to invaders.

My results also indicated that invasive species are “leafier,” investing more heavily in leaf tissue at the expense of stems and roots. In some phylogenetically controlled studies in low-resource environments, invaders have been shown to invest more than non-invaders in root mass [45], [46], but in this case, invaders allocated less biomass to root mass. Investing in additional biomass to more thoroughly mine the soil might be adaptive in a low-nutrient environment, but my results suggest invaders compensate with other efficiencies. For instance, invaders were more photosynthetically nitrogen-use efficient–able to photosynthesize on less leaf N–as well as more efficient at taking up nutrients across the root surface (SAR_N_ and SAR_P_). Photosynthetic nutrient-use efficiency frequently correlates positively with SLA and negatively with DMF, because denser leaves with thicker cell walls have lower internal conductance and may require more N allocation to structural proteins rather than the photosynthetic apparatus [47], [48]. Several investigators have associated higher PNUE with invaders in phylogenetically constrained pairings of invaders and non-invaders [41], [49], [50]. Water-use efficiency, a trait that has been shown to trade off with PNUE [51], was higher in non-invaders. A few empirical studies have recorded either higher or lower WUEi in invaders when congeneric pairs are compared [43], [49], [50], suggesting that its relationship with invasiveness may depend on the environment invaded.

### Plasticity

Contrary to expectation, no significant plasticity differences between invaders and non-invaders were found for individual traits, nor for groupings of traits. However, all of the groupings, and all but two of the individual traits, showed (nonsignificant) trends of higher plasticity in invaders. The plasticity indices calculated here are based on species mean values, so this result may be partly owing to a lack of statistical power in a study with only 10 species to compare. However, the trait value analysis (above) was also performed on species mean values, but resulted in nearly all the traits being highly significantly different between invaders and non-invaders. Therefore plasticity differences related to invasion status are much weaker than trait value differences.

Other studies have used meta-analysis to harness greater statistical power in answering this question, although most of the studies they synthesize compared invaders to native species, not necessarily non-invasive ones. The results have been mixed. One, a meta-analysis of invasive-native pairs [13], concluded that invaders showed greater plasticity overall; it also identified six individual traits for which invaders were significantly more plastic. Of these traits, three have analogs in the present study (PNUE, WUEi, and root:shoot ratio) but were not found to be more plastic in invasive pines. Four other traits represented in both studies (N content, P content, photosynthesis, and SLA) exhibited no plasticity relationships to invasiveness in either study. A second recent meta-analysis [12] covered some of the same papers, but restricted the trait differences studied to a narrower range of environmental conditions (e.g., SLA plasticity was only examined in studies where light was a variable). It found that there was no general trend of higher plasticity in invaders, and individual trait plasticities were not reported. The authors reached the conclusion that invaders' success must be due more to constitutive factors–i.e., trait values–than to trait plasticity, or else that higher plasticity is only characteristic of invaders in the early phase of invasion, and is gradually eroded by genetic assimilation [12]. A third meta-analysis [52] found that more widely distributed alien species had higher plasticity than less-widespread aliens in the response of biomass to increases of light, water, and nutrients, but that this plasticity trend did not extend to the individual traits of SLA or root:shoot ratio. In sum, the evidence for greater plasticity in particular functional traits is weak even when large numbers of species comparisons are considered.

### Relationship between performance and trait values or trait plasticity

As a group, invasive pines in this study clearly outperformed their non-invasive congeners in response to an increase in N supply, growing at a slightly higher rate when N was low, but nearly twice as fast when N was high. This significant phenotype-by-environment interaction is evidence that invasives conform to a “Master-of-some” strategy, i.e., a superior ability to increase fitness in response to a favorable environment [15]. Several previous studies of pines have found that invasive pines have higher RGR than non-invasive ones [46], [50], but this result indicates that invaders may be most advantaged in high-resource environments.

Godoy and colleagues [21] suggested two mechanisms, not mutually exclusive, for explaining how functional traits might underlie invasives' superior fitness gains (or smaller fitness losses) in a changing environment: 1) higher trait plasticity in invaders that results in greater fitness than non-invaders; and 2) similar levels of plasticity among both groups, coupled with constantly superior values for fitness-related traits in invaders. Evidence to support the first mechanism can come from regressions of trait plasticity against fitness [13], [20]. For pines in this study, only one trait showed a significant correlation between its plasticity and the performance measure RGR, indicating that, in general, higher plasticity in functional traits is not the best explanation for the performance response observed. Moreover, the biological significance of the sole performance-plasticity correlation (for SMR) is called into question, first by the existence of an influential outlier whose removal decreased its statistical significance, and second by the fact that SMR trait values themselves were not significantly correlated with RGR. This reveals one of the drawbacks of using a plasticity index in the correlation–namely, plasticity is represented by any change, whether an increase of decrease. For SMR, both invasives and non-invasives sometimes increased and sometimes decreased the stem mass ratio when N supply (and growth rates) increased. This makes SMR a poor indicator of growth rate and a poor candidate for an important functional trait, but because the size of the change in either direction is generally larger for invaders and smaller for non-invaders, the plasticity index itself is correlated with performance. In short, there is little evidence from regressions of individual trait plasticities against RGR to support the contention that invaders have higher adaptive plasticity.

Nonetheless, if invaders grow faster at higher N, there must be some underlying trait plasticity that explains why. One clue comes from regrouping the plasticity indices and correlating a combined set of traits with RGR, to see what general categories of plant function may have the greatest adaptive plasticity. There was a significant plasticity-performance correlation for the grouping of traits related to leaf structure and metabolism, whose combined plasticity explained ∼84% of the variance in RGR, but not for the other groupings. Another way of answering the question is to look for traits where both the trait value itself and its plasticity index maximize their relationship with RGR. Traits for which the plasticity index and the trait value itself each explain at least half the variation in RGR in this study (i.e., both *r* >50) are the biomass allocation trait RMR, the whole-plant trait SAR_P_, and the leaf-level traits SLA, N_area_, A_area_, and g_s_. These two lines of evidence suggest that, on the whole, traits related to leaf chemistry, morphology, and photosynthetic ability are the best candidates for adaptively plastic traits in these species–with the caveat that this study only considered differences in N supply, and that other gradients might have produced a different suite of traits. It is also possible that the differential RGR response of invaders is due to plasticity in traits not measured here.

Overall, though, the relationship between functional trait plasticity and performance in this study is not strong. Some traits instead conform to the second possible mechanism for higher fitness–consistently superior values in invaders of traits that either lack plasticity, or have lower plasticity in invaders than non-invaders [21]. The traits LAR, LMR, SLA, and PNUE all fit this description. Contrary to the idea of objectively superior plasticity conferring invasion success, Godoy and colleagues posit the existence of a “general purpose phenotype,” characterized by high mean values of fitness-related traits coupled with sufficient plasticity to compete in a wide variety of environments. This phenomenon may explain the results of other multispecies invasive-noninvasive comparisons that found invaders' higher plasticity in biomass production unaccompanied by higher plasticity in presumably related functional traits [8], [53] as well as the mixed results from the various meta-analyses [12], [13], [52].

It is important to note, too, that fast growth, the fitness proxy here, is not necessarily the best strategy in every environment [13]; in low-resource systems, building structurally sound or heavily defended tissues may be more important in the long run than high RGR [49]. Also, the use of commercially sourced, rather than wild-sourced, seeds may have introduced a bias toward faster growth that is not representative of introduced pine populations. Other limitations of the study include ignoring reproductive traits like seed mass or fecundity that have proved to be powerful predictors of invasion success in pines and other woody species [54], and failing to account for phylogenetic distances among pairs of species, as well as potential differences among invaders in niche breadth and the width of distribution in their invaded ranges. However, this work provides a rare picture of the comparative functional significance of plant traits and plasticity that is lacking in many invasive-noninvasive comparisons. Future work on this topic could expand the range of traits and environments studied, especially with regard to mimicking probable future conditions under climate change, and comparing traits and fitness proxies between invaders and co-occurring natives in the invaded range.

## Supporting Information

Table S1 **Mean values for functional traits by species in each nutrient treatment.**
(XLS)

Table S2 **Relative trait range values (plasticity indices) for functional traits by species.**
(XLS)
