# Correction: Trait Values, Not Trait Plasticity, Best Explain Invasive Species' Performance in a Changing Environment

**DOI:** 10.1371/annotation/0ed08a64-3742-4f95-9695-88b42d216d18

**Published:** 2013-05-03

**Authors:** Virginia Matzek

Due to an error during the production process, tables 2 and 4 were misprinted. Correct versions of the tables can be viewed 

**Figure:**
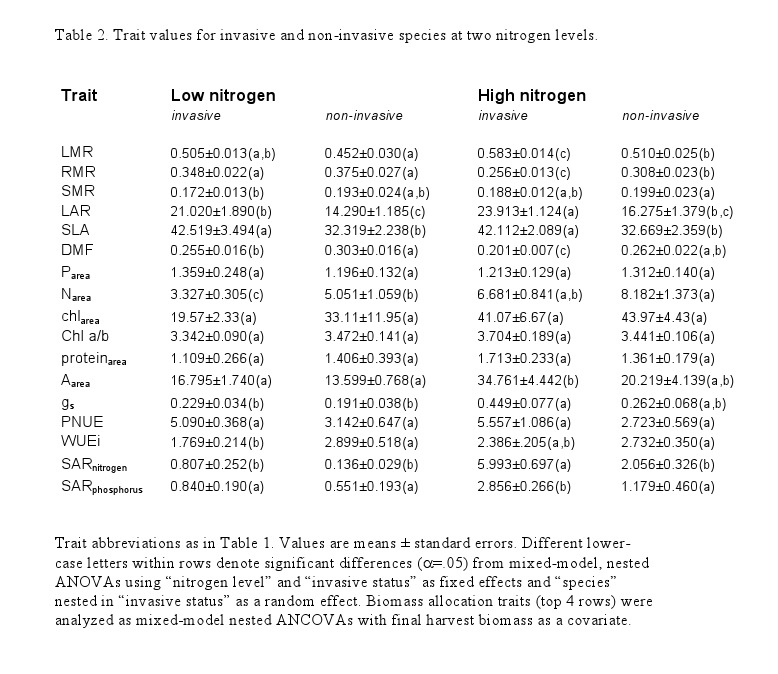


and 

**Figure:**
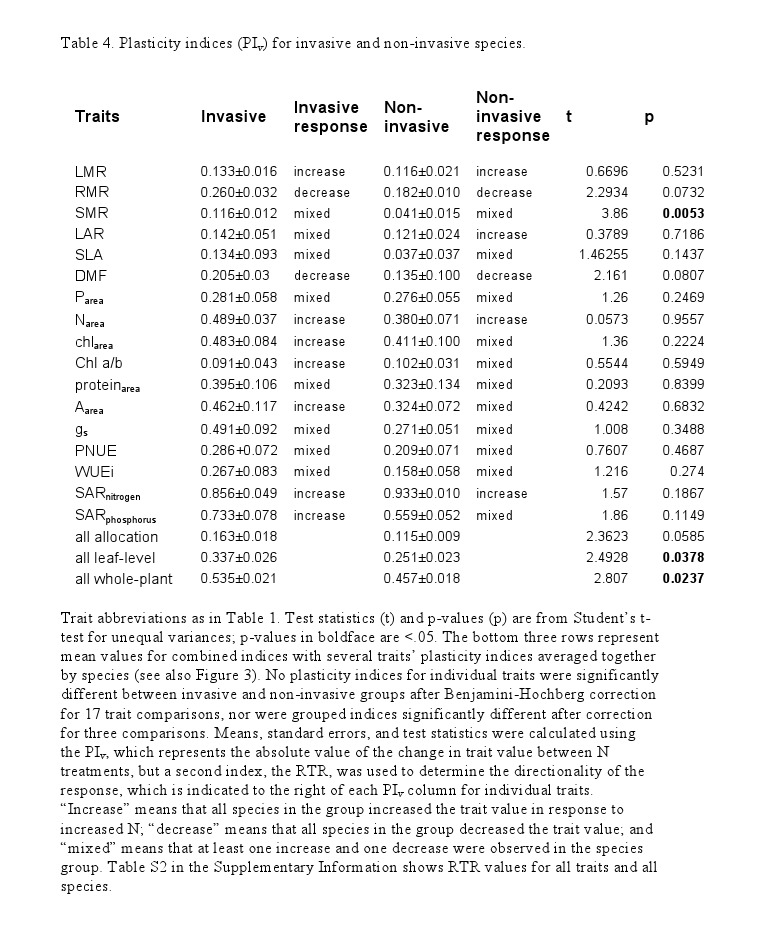


. 

